# Evaluation of dental students' learning curve in intraligamentary anesthesia using different syringe systems: A prospective crossover study

**DOI:** 10.1002/jdd.13754

**Published:** 2024-10-29

**Authors:** Philipp Luhrenberg, Mirjam Renders, Diana Heimes, Anke Hollinderbäumer, Sebahat Kaya, Solomiya Kyyak, Saskia V. Schröger, Daniel G. E. Thiem, Helen Wagner, Peer W. Kämmerer

**Affiliations:** ^1^ Department of Oral and Maxillofacial Surgery, Facial Plastic Surgery University Medical Center, Johannes Gutenberg University Mainz Mainz Germany

**Keywords:** academic training, CCLAD, clinical skills, dental anesthesia, educational, intraligamentary anesthesia

## Abstract

**Purpose/objectives:**

This prospective crossover preclinical trial aimed to evaluate the learning curve of dental students in successfully administering intraligamentary anesthesia (ILA) using three different syringe systems.

**Methods:**

Dental students performed ILA using three devices in two separate sessions, each targeting mandibular and/or maxillary premolars. The devices included two manual systems (pistol‐type and lever‐based) and one computer‐controlled local anesthetic delivery system (CCLAD). The primary research parameter was the success rate of anesthesia, defined as the percentage of successful ILA administrations confirmed by a negative response to a cold test. Secondary parameters included pain experienced during needle penetration and injection, students' self‐reported levels of mental tension and handling of the syringes, and any potential side effects.

**Results:**

A total of 110 students performed ILA on 599 teeth during the study period. When comparing the CCLAD system to the manual syringes, the CCLAD system exhibited a significantly higher overall success rate in the first session (92.5% vs. 77.4%; *p* < 0.001), potentially due to its precise control of anesthetic flow and pressure, which likely facilitated more effective anesthetic delivery. However, when examining the individual manual techniques, no significant difference was found between the pistol‐type manual and the CCLAD system (*p* = 0.66). All techniques' success rate increased from the first to the second session (80.4% vs. 86.9%; *p* = 0.0357). Additionally, penetration pain demonstrated a significant decrease across all techniques (*p* < 0.01). Notably, students' anxiety levels decreased, and self‐assurance increased significantly over the sessions. Undesired reversible side effects were documented in 10.9% of cases.

**Conclusion:**

These findings suggest that repeated practice of ILA, particularly with different syringe systems, enhances anesthetic success and psychological readiness for patient interaction. Additional training sessions may further improve proficiency.

## 1 INTRODUCTION

Understanding the learning curve is essential in educational contexts as it offers valuable insights into learning efficiency and the progression of skill acquisition. This curve illustrates the relationship between the effort invested in learning tasks and subsequent achievement, which can be gauged by factors such as time taken or the number of attempts required to attain proficiency.^1^ Typically depicted graphically, the learning curve begins at zero, ascends from high‐gradient to low‐gradient areas, and eventually plateaus at an asymptotic maximum.^1^, ^2^ The steepness of this gradient varies depending on factors such as the complexity of the learning content or skill, learners' starting points, interests, preferred learning styles, the learning environment, and the efficacy of training methodologies.^3^, ^4^

In dental education, students acquire technical skills through preclinical and clinical courses while honing their soft skills through kinesthetic learning modalities. Although the learning curve has been explored in dental education, particularly in areas such as implantology and digital intraoral scanning for young professionals,^5^, ^6^ its investigation regarding local anesthesia, particularly intraligamentary anesthesia (ILA), remains scarce in the literature. While the learning curve is a valuable tool for self‐reflection (e.g., in tracking the progress, identifying strengths and weaknesses, setting realistic goals, enhancing self‐efficacy, facilitating feedback and reflection, and improving educational strategies) in various surgical procedures within oral and maxillofacial departments,^7^, ^8^, ^9^ there is a notable gap in evidence regarding its application to ILA in dental education. Factors influencing the learning curve include the number of attempts, the quality of feedback, the presence of didactic reviews, and the integration of theoretical knowledge with practical skills. Didactic sessions that include lectures, demonstrations, and reviews of theoretical concepts can reinforce the practical skills acquired during hands‐on sessions. These reviews help solidify understanding, address common mistakes, and introduce advanced techniques, enhancing learning.^10^, ^11^

**FIGURE 1:**
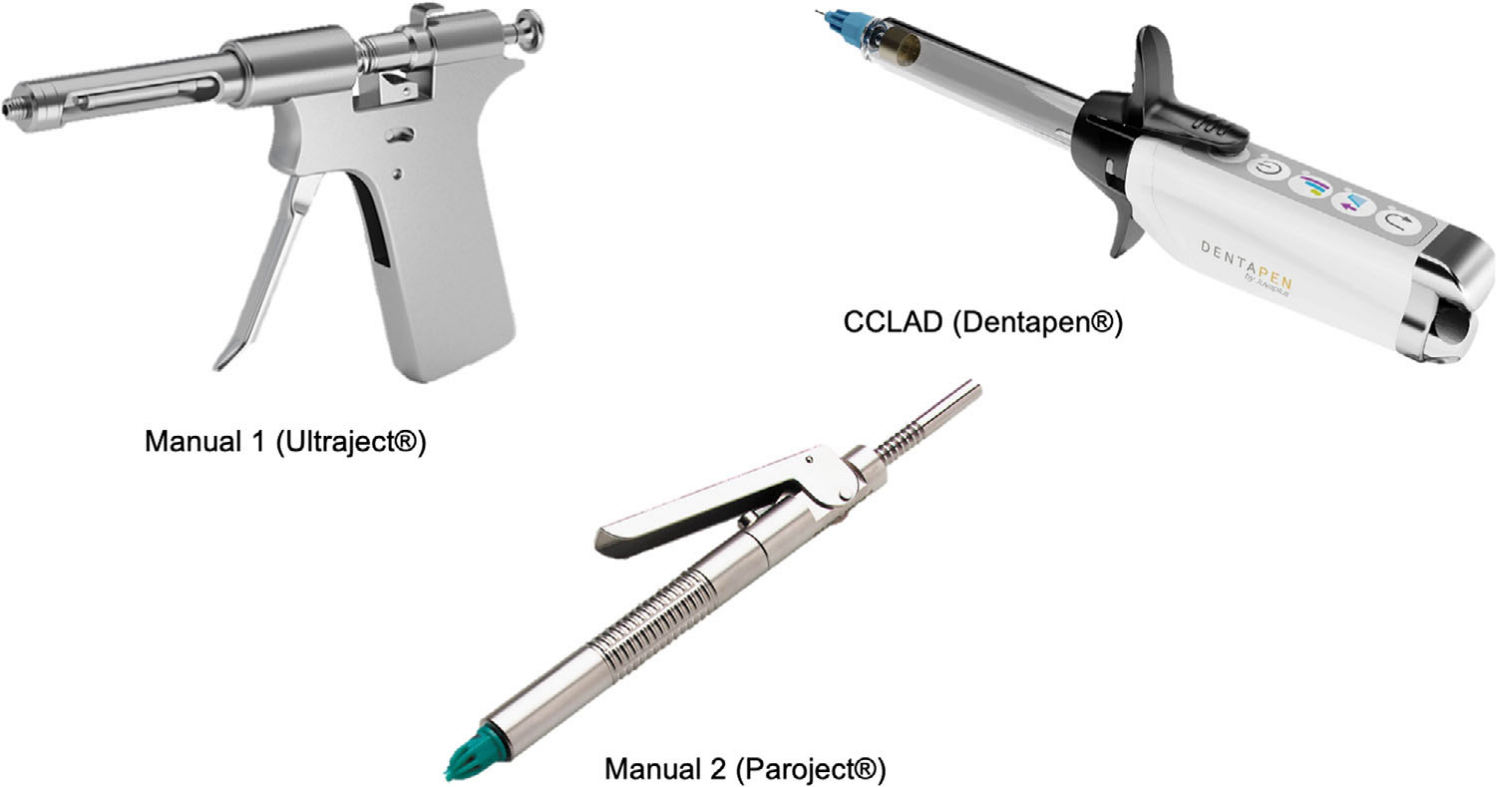
Syringe systems that were utilized in the study. Images provided by the respective manufacturers: Septodont, RØNVIG Dental Mfg., A/S, and Juvaplus. CCLAD, computer‐controlled local anesthetic delivery system.

ILA represents a rapid and effective method for achieving anesthesia in a single tooth without involving the surrounding soft tissues such as the lips, cheeks, and tongue. During ILA administration, a short 30‐gauge needle is inserted into the periodontal ligament (PDL) until reaching the alveolar border. After PDL penetration, approximately 0.2 mL of anesthetic solution is delivered for each root.^12^, ^13^ Due to the PDL's significant resistance, approximately four times higher than palatal tissue and 30 times higher than vestibular mucosa, specialized high‐pressure syringe systems are recommended.^14^ Manual syringe systems come in various designs, offering slow, nontraumatic injections with and without pressure limits to mitigate potential side effects such as pain from high pressure and postoperative tooth elongation sensation.^15^ Computer‐controlled local anesthetic delivery systems (CCLAD) have shown promise in reducing injection pain during dental local anesthesia (Figure 1).^16^ However, they may require more clinical experience than manual syringes.^17^ Additionally, potential drawbacks of CCLADs include their design, weight, cartridge location, aspiration capability, and the costs associated with special consumables. In addressing these concerns, a novel CCLAD device (Dentapen®, Juvaplus; Figure 1) offers a lightweight, cordless design with aspiration capacity using standard needles and anesthetic cartridges. In its ILA mode, this device provides the slowest speed at maximum force, with a gradual to constant flow of anesthetic.^18^

It has been observed that ILA is challenging to administer, characterized by a relatively flat learning curve, requiring many attempts or experiences to achieve proficiency.^17^ In a European survey, dental students expressed a need for preclinical training in local anesthesia, particularly in ILA.^19^ However, the optimal number of training sessions required and the most effective syringe system for ILA remain to be determined. Therefore, this crossover study sought to explore students' learning curves while performing ILA in a preclinical setting across two consecutive training sessions. Our study primarily aimed to assess the learning curve of dental students in administering ILA using three different syringe systems, with the success of anesthesia as the primary research parameter. Additionally, we aimed to compare the effectiveness of three different syringe systems in facilitating skill development and proficiency.

## 2 MATERIALS AND METHODS

This crossover trial was conducted at the Department of Oral, Maxillofacial, and Plastic Surgery of the University Medical Centre Mainz, Germany, following approval from the local ethics committee of Rhineland‐Palatinate (No. 837.042.10 (7051) and (2019‐14177‐NIS)) and following the principles outlined in the Declaration of Helsinki. Dental students enrolled in the third year of study and participating in the “Surgical Propaedeutics” course were eligible for inclusion. Exclusion criteria comprised contraindications to local anesthetics or their constituents, lack of compliance, and refusal to participate.

### 2.1 Materials

ILA was administered using two manual systems: pistol‐type manual 1 (Ultraject®, Septodont) and lever‐based manual 2 (Paroject®, RØNVIG Dental Mfg. A/S), as well as a CCLAD (Dentapen®, Juvaplus; Figure 1 (pictures obtained from manufacturer). The local anesthetic used for injection in all three systems was Ultracain D‐S (articaine 40 mg/mL plus adrenaline 1:200,000 [0.006 mg/mL], Septodont).

### 2.2 Methods

All participants were briefed about the study 1 week before commencement and received theoretical instructions, including a 1‐h lecture and a clinical video demonstrating the administration of ILA. Additionally, students underwent training in various dental local anesthesia techniques (i.e., infiltration, nerve blocks, and ILA with the three different syringe types) under the supervision of dentists, utilizing human cadavers at the Institute of Functional and Clinical Anatomy, University Medical Center Mainz.

Upon consenting to participate, students formed pairs and voluntarily administered ILA to each other. Each student performed and received ILA on three teeth per session, including mandibular and/or maxillary premolars. During each session, participants utilized each syringe system once to administer ILA. The study was conducted over three semesters, with each semester having a new group of students. Each student participated in two sessions conducted 2 weeks apart within the same semester. Therefore, each student had a total of two hands‐on sessions. The sessions were spread out as follows:

Semester 1: Group A (approximately 37 students) had two sessions, 2 weeks apart.
Semester 2: Group B (approximately 36 students) had two sessions, 2 weeks apart.
Semester 3: Group C (approximately 37 students) had two sessions, 2 weeks apart.

Each group completed their training within one semester. The sessions were structured to ensure that each student had equal opportunities for practice and feedback within their respective groups.

Before ILA administration, the vitality of the target tooth was confirmed using a cold spray applied to a cotton pellet. Subsequently, students performed ILA on each other, injecting approximately 0.2 mL of local anesthetic at a second premolar's mesial and distal sites. Pulpal anesthesia was evaluated 10–20 s postinjection using cold spray on a cotton pellet (sufficient: no feeling during cold test, insufficient: feeling during cold test). Pain levels associated with needle penetration and local anesthetic injection were assessed using an 11‐point segmented numeric rating scale (NRS). For the evaluation of secondary study parameters, participants self‐assessed their mental tension using a five‐point Likert scale (ranging from very nervous [5] to very relaxed [1]). They rated the ease of system handling (ranging from very difficult [5] to very easy [1]; Table 1).

**TABLE 1:** List of questions asked of students (category, question, and scale used).

Category	Question	Scale
Pain experienced during needle penetration	“Rate the pain experienced during needle penetration.”	11‐point numeric rating scale (0–10)
Pain experienced during injection	“Rate the pain experienced during the injection of the anesthetic.”	11‐point numeric rating scale (0–10)
Mental tension	“How nervous did you feel while administering the anesthesia?”	5‐point Likert scale (1–5)
Ease of handling	“How easy did you find the handling of the syringe system?”	Five‐point Likert scale (1–5)
Side effects	“Did you experience any side effects such as pain, inflammation, or a sensation of tooth elongation?”	Yes/No
Side effects description	“If yes, please describe the side effects you experienced.”	Open‐ended

The second session was conducted 2 weeks later, following the aforementioned procedure. This entire process was repeated two times, corresponding to three different semesters, allowing for comparing data between the first and second sessions to reconstruct a comprehensive learning curve. After each session and 1 week after the respective session, any side effects or post‐ILA discomfort experienced by participants were carefully documented.

### 2.3 Specific influences on the learning curve in our study

ILA is a complex skill where the amount and pressure must be precisely controlled, and anatomy must be clearly understood. Further, it is a hands‐on skill that requires the student to develop cognitive and motor skills. The content design was such that students' skills were built sequentially over the two sessions. The students were in the third year of dental studies. Their background consisted of diversified exposure to techniques of local anesthesia. This difference in baseline levels probably affected the initial performance and learning rates. However, the standardized training gave all participants a similar platform. The students' intrinsic interest in mastering the clinical skills, together with their preferred learning styles, was catered for by the different modes of teaching, namely, lectures, video demonstrations, and hands‐on practice. During such sessions, feedback from peers and tutors played a vital role in fine‐tuning students' techniques. This multimodal approach helped cater to different learning preferences and enabled better content absorption. The controlled preclinical environment provided a conducive learning environment without the pressures of actual patient care. Thus, the students could focus on acquiring skills without additional pressure due to clinical performance, an added advantage to the learning curve. In between the two sessions, a feedback system about their performance will help the students realize areas of improvement. This iterative process of practice and review was indispensable in building their skills and lessening their anxiety levels in later sessions.

### 2.4 Statistics

Student's *t*‐tests were employed for *p* values less than 0.05, while Mann–Whitney *U*‐tests for independent samples were used for *p* values greater than 0.05. The influence of nominal variables was assessed through chi‐square tests and cross tables, with a predetermined global significance level of *p* ≤ 0.05. Statistical significance was assumed solely for the primary research parameter. Descriptive reporting was used for all other *p* values. All statistical analyses were performed using GraphPad Prism 10 (Version 10.2.3, Insight Partners, GraphPad Holdings).

## 3 RESULTS

### 3.1 Study population and number of injections

One hundred ten students participated in the study between April 2019 and February 2023, spanning three semesters, with a response rate of 100%. The mean age of the students performing ILA was 22.5 years. Female participants were more prevalent, comprising 74 out of 110 students, while male participants totaled 36. Before ILA administration, each tooth designated for anesthesia responded positively to the sensibility test. For session 1, data for a total of 301 teeth were available for analysis (manual 1: *n* = 90, manual 2: *n* = 105, CCLAD: *n* = 106), and for session 2, a total of 298 teeth were analyzed (manual 1: *n* = 100, manual 2: *n* = 98, CCLAD: *n* = 100).

## 4 PRIMARY RESEARCH PARAMETERS

### 4.1 General success rate

**FIGURE 2:**
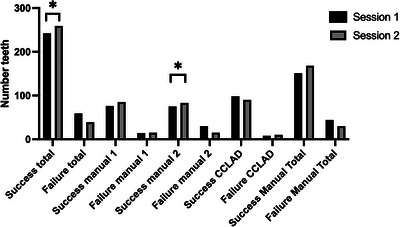
Bar charts showing the results for the success rate of intraligamentary anesthesia (ILA; * stands for statistically significant differences). CCLAD, computer‐controlled local anesthetic delivery system.

During the initial session, ILA procedures were performed 301 times, resulting in 242 instances where teeth exhibited negative sensibility postadministration, yielding an overall success rate of 80.4%. Remarkably, a significant increase in the success rate was observed during the subsequent session, with 259 out of 298 teeth demonstrating negative sensibility after ILA administration, reflecting an improved success rate of 86.9% (*p* = 0.0357; Figure 2).

### 4.2 Success concerning the syringe system

During the first session, manual devices exhibited lower success rates than the CCLAD system (98/106; 92.5% vs. 151/195; 77.4%; *p* < 0.001). However, the Paroject® syringe system (manual 2) showed significantly diminished success rates compared to both the Ultraject® (manual 1) system (75/105; 71.4% vs. 76/90; 84.4%; *p* = 0.0388) and the CCLAD syringe (*p* < 0.001). Also, there was no significant difference in a single comparison of the CCLAD and the manual 1 syringe (*p* = 0.066). In the subsequent session, there was no significant difference in terms of anesthetic success between CCLAD (90/100; 90%) and manual systems (168/198; 84.8%) anymore (*p* = 0.281). Additionally, there was no difference between manual 1 (85/100; 85%), manual 2 (83/98; 84.7%), and CCLAD anymore (manual 1 vs. manual: *p* > 0.99; manual 1 vs. CCLAD *p* = 0.39; manual 2 vs. CCLAD *p* = 0.291). When comparing the success rates between sessions, it was observed that only the success rate of the manual 2 system significantly improved (*p* = 0.028; Figure 2).

## 5 SECONDARY RESEARCH PARAMETERS

### 5.1 Penetration pain and injection pain

**FIGURE 3:**
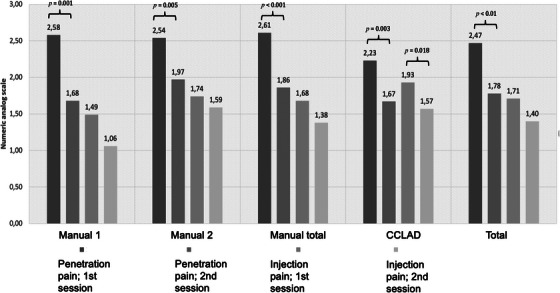
Bar chart showing penetration and injection pain for intraligamentary anesthesia (ILA). *p* values are given for statistically significant differences only.

The mean cumulative score for penetration pain, assessed via the numeric analog scale, exhibited a significant reduction in the second session compared to the first (first session: 2.47 ± 1.83; second session: 1.78 ± 1.48; *p* < 0.01); this trend was consistent across all syringe devices (Figure 3). In intergroup analyses, notably lower penetration pain was observed with the CCLAD compared to the manual 2 system (*p* = 0.049). For injection pain, a significant decrease was observed between the first and second sessions for the CCLAD system only (*p* = 0.018; Figure 3).

**FIGURE 4:**
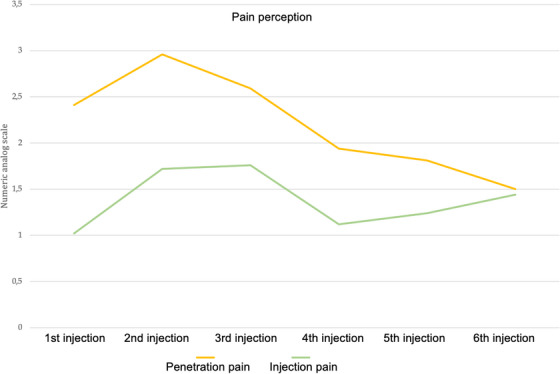
Line diagram showing the penetration and injection pain depending on the sequence intraligamentary anesthesia (ILA) was administered.

The type and sequence of syringes used did not significantly change the outcome of the pain levels reported. In general, pain values generally followed a pattern of starting low, peaking during the second and third injections, and declining steadily, particularly for penetration pain (Figure 4). Nonetheless, there was an overall reduction in penetration pain from the second to the final injection (*p* = 0.03).

### 5.2 Evaluation of ILA

**FIGURE 5:**
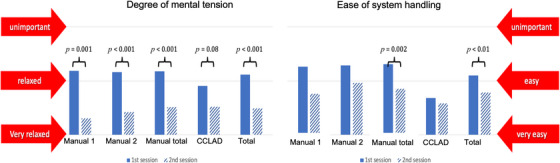
Bar charts show the level of mental tension and the subjective ease of handling concerning the different syringe systems of the two consecutive sessions. *p* values are given for statistically significant differences only. CCLAD, computer‐controlled local anesthetic delivery system.

Following the administration of ILA, students provided immediate feedback on their experience. The consensus among students was a notable reduction in mental anxiety during ILA administration in the second session (*p* = 0.002), a trend observed across all syringe systems (Figure 5A). Additionally, students reported heightened confidence in utilizing all syringe systems during the second session, with statistically significant improvements noted for the manual 1 system (*p* = 0.022; Figure 5).

### 5.3 Adverse effects

Among the participating students, undesired side effects were reported 62 times, accounting for 10.9% of all cases. The most common side effect, reported in 46 instances (74.2%), was pain from slight inflammation surrounding the treated teeth. Ten students (16.1%) experienced a sensation of elongation at the site of ILA administration, while five cases (8.1%) exhibited reversible discoloration of the anesthetized papilla. One case (1.6%) was also noted with general aphthosis, potentially associated with ILA administration. Importantly, no statistically significant association was found between the occurrence of adverse effects and the specific syringe systems utilized (all: *p* > 0.05).

## 6 DISCUSSION

This study investigated ILA success obtained by dental students across multiple sessions using three syringe types. The findings indicate a notable enhancement from the initial to the subsequent session, giving evidence of the beneficial impact of repeated injections. In brief, the overall success rate of ILA increased from the first to the second session, and students reported reduced pain and anxiety levels. However, the study was limited to only two sessions, primarily showing initial improvement (“learning curve”) rather than achieving proficiency. The learning curve represents the relationship between the effort invested in learning a skill and proficiency achievement. In the context of our study, the learning curve was assessed by evaluating the success rate of ILA administration, pain experienced during needle penetration and injection, and students' self‐reported levels of mental tension and confidence. By measuring these parameters across two sessions, we aimed to capture the initial stages of the learning process and the improvements associated with repeated practice. The number of attempts required to achieve true proficiency—defined as consistent success with minimal pain and anxiety—remains undetermined, and additional sessions would likely provide more comprehensive insights.

Indeed, ILA is commonly considered a supplementary technique, particularly when conventional methods like the inferior alveolar nerve block (IANB) fail to achieve the desired outcomes.^20^ While this study did not specifically aim to evaluate this, our findings suggest that ILA can be a valuable adjunctive approach in such scenarios. Clinicians may sometimes use only ILA, depending on the clinical situation, and our results reinforce this potential utility of ILA. As students progress along the learning curve and attain proficiency in ILA administration through the repeated practice provided by the two sessions, they enhance their technical skills and bolster their confidence in employing this technique.^21^, ^22^, ^23^, ^24^

Recent studies have underscored ILA's comparable or even superior efficacy compared to IANB. However, the correlation between the success of these anesthesia techniques and the actual procedures being performed, and the specific teeth involved needs to be clarified.^12^, ^13^, ^15^, ^25^ Considering the advantages of ILA, this technique should be trained before attempting successful application. Compared to similar studies, the sample size in this study is relatively large. When administering primary ILA, success rates typically range from 69% to 100% when performed by experienced physicians.^26^, ^27^, ^28^ In the present study, success rates achieved by students without prior patient treatment experience were approximately 80% or even higher, showing similar results to those of experienced dentists. Higher failure rates in the initial session are expected when untrained and inexperienced students perform ILA, often referred to as “first‐time users.” Our study demonstrates that gradual learning of ILA with adequate preparation (such as lectures and practical training on human cadavers) and multiple injections at different times can enhance students' performance.^17^ Given the learning curve, it is plausible that the frequency of successful ILA administrations will increase in routine practice. However, it is important to note that our results may be limited because the surrounding tissues of the teeth (gingival mucosa, PDL, and periapical region) were healthy and noninflamed, potentially making anesthesia more challenging.^20^, ^29^, ^30^, ^31^ Furthermore, it is worth noting that ILA was performed on the second premolar rather than molars, which are generally considered more challenging to anesthetize, particularly the lower first molar.^27^ Third, it is essential to acknowledge that sufficient anesthesia was solely assessed using cold spray without further intervention to confirm pulpal anesthesia. This limitation may impact the applicability of ILA for restorative treatments or minor surgical procedures, such as extractions or osteotomies, in both primary and permanent dentition.^13^, ^32^

Using different syringe systems in teaching ILA benefits skill development in several ways. The initially high success rate with the CCLAD system can be attributed to several factors. The CCLAD system allows for very accurate control of the flow rate and pressure of the anesthetic solution; this ensures a more uniform and effective delivery of the anesthetic. This precision minimizes possible anesthetic leakage and guarantees that the anesthetic will be delivered directly into the PDL space, thereby increasing the overall effectiveness of anesthesia. Also, its ergonomic design and automation of features lower operator fatigue with better handling due to minimal efforts required in this process, especially in the case of students still acquiring their hand skills. The feedback mechanisms of the CCLAD system provide real‐time adjustments, resulting in more accurate delivery and fewer variables compared with manual injections.^33^, ^34^ Manual systems, on the other hand, might require students to develop fine motor skills and manual dexterity, which are essential for effective anesthesia administration. Students comprehensively understand the techniques and skills required for different clinical scenarios by practicing with both systems.

A study conducted by Kämmerer et al. used a differently designed CCLAD (STA®) and compared it with a manual device, resulting in lower success rates than the manual devices.^26^ However, it is important to note that the two CCLADs exhibit significant design and user interface differences. The Dentapen® utilized in our investigation is a pen‐shaped device (Figure 1) activated simply by pressing the black lever on the handpiece. In contrast, the STA® requires activation via a footswitch, a feature that may require some degree of familiarization for optimal use. Additionally, it was observed that the success rates with the Dentapen® slightly decreased by approximately 3% in the second session. This decline could be attributed to technical issues and, in some instances, incorrect device settings. Significant differences in the anesthetic success between the pistol‐type and lever‐type manual systems might be attributed to the more ergonomic design and enhanced control features of the former, making it easier for students to achieve effective anesthesia on their first attempt. However, there is no data in the literature to substantiate this result. Additionally, no such differences were observed in the second session. This leads to the conclusion that manual syringes, or a CCLAD, are suitable devices for ILA.

Regarding pain perception, many previous studies have not differentiated between penetration pain and injection pain, often combining them into a single cumulative pain variable. However, compared to studies conducted by experienced professionals, the level of discomfort experienced during ILA in our second session remained comparable.^15^, ^17^, ^35^, ^36^ However, an increase in injection pain during the fifth and sixth injections was detected, which might be directly attributed to the learning curve as the initial injections might have been performed more cautiously. In contrast, later injections might have been executed with more force or less precision due to overconfidence or haste, resulting in increased pain. Second, a variation in technique might be assumed as students became more familiar with the procedure. On the contrary, for penetration pain, a steady decrease was detected.

Students who administered ILA to each other exhibited initially low average anxiety levels. However, through repeated practice sessions, anxiety levels decreased from “relaxed” (2.1) to “very relaxed” (1.4). This reduction in mental tension and anxiety echoes discussions in several studies. For instance, Sánchez‐Garcés et al. demonstrated a gradual decrease in anxiety levels among their students before, during, and after administering the IANB to each other. This anxiety reduction correlated with increased confidence in handling real‐life scenarios, recognizing landmarks, and determining insertion points.^37^ Other researchers have noted a reverse correlation between anxiety levels and students' self‐confidence.^38^, ^39^ These findings underscore the importance of addressing anxiety through appropriate training and support mechanisms to enhance students' confidence in performing clinical procedures such as ILA.^21^, ^22^, ^23^, ^24^

In the second session, all participants demonstrated increased confidence in navigating the different syringe systems. While the CCLAD yielded the most favorable outcomes, notable improvement was observed with the manual devices, indicating significant learning progress, which is crucial for sustaining motivation in the learning process. Trainees frequently reported that using mechanical devices enhanced their understanding of ILA techniques. These devices offer valuable haptic feedback regarding the pressure required while assessing for indicators such as ischemia in fixed soft tissue, backflow fluid as an adverse sign, and painful facial reactions, enabling students to adjust the pressure on the lever accordingly. Therefore, mechanical devices may facilitate a deeper acquisition and training of the technique for educational purposes.

While ILA is generally considered a safe technique for shorter and minor interventions, it is not entirely complications‐free. Fortunately, permanent damage following ILA has not been reported in the literature. Also, our study only documented temporary complications in 62 cases (10.9%), primarily manifesting as discomfort or pain. Consistent with findings from comparable studies, patients typically experience mild postoperative pain, with moderate or severe forms being rare and reversible.^40^ The available data on the incidence of postoperative pain are heterogeneous, with rates reported to reach as high as 31%.^36^, ^40^ After ILA, moderate soft tissue inflammation is observed in the histological appearance within 24 h. Signs of inflammation disappear entirely over time, and the junctional epithelium migrates apically into the notch, likely due to the needle tip positioning in the sulcus.^38^, ^41^ Nevertheless, complications following ILA are generally less severe compared to those after IANB, which may lead to issues such as trismus and temporary or even permanent nerve injury.^42^, ^43^

Teaching dental anesthesia has long been a subject of debate. According to students, various techniques, particularly ILA, should be extensively trained in a preclinical setting.^19^ As demonstrated in the present study, initial training on human cadavers allows students to focus on refining their technique without the interference of mental tension, thus helping to prevent mistakes.^4^, ^44^ Alternatively, training without the presence of patients can be conducted using haptic simulation models or virtual reality simulators.^45^, ^46^ However, it was critically reflected that haptic simulations might sometimes depict distorted human anatomy, potentially leading to lower success rates.^47^ “Real‐life training,” initially conducted among students, appears to be the most widely accepted method before performing local anesthesia on patients.^39^, ^48^

## 7 CONCLUSION

Incorporating multiple ILA sessions and utilizing various syringe systems into preclinical dental education curricula could improve student competency in local anesthesia administration. This approach enhances success and better prepares students for patient care. Future research should focus on extending the training period to capture the entire learning trajectory and provide more definitive answers regarding attaining proficiency in ILA.

## AUTHOR CONTRIBUTIONS

All authors made substantial contributions to the conception and design of the manuscript. Philipp Luhrenberg, Mirjam Renders, and Peer W. Kämmerer performed the literature search. All authors interpreted the data. All authors drafted the work and revised it critically for important intellectual content. All authors agree to be accountable for all aspects of the study design and its content. All authors approved the final submitted version.

## CONFLICT OF INTEREST STATEMENT

The authors declare no conflicts of interest.

## PATIENT CONSENT STATEMENT

Informed consent was obtained from all participants. All methods were carried out by the Declaration of Helsinki.

## Data Availability

All data generated and analyzed during this study are included in this published article.

## References

[1] Pusic MV , Boutis K , Hatala R , Cook DA . Learning curves in health professions education. Acad Med. 2015;90(8):1034‐1042. 25806621 10.1097/ACM.0000000000000681

[2] Pusic MV , Boutis K , Pecaric MR , Savenkov O , Beckstead JW , Jaber MY . A primer on the statistical modelling of learning curves in health professions education. Adv Health Sci Educ Theory Pract. 2017;22(3):741‐759. 27699508 10.1007/s10459-016-9709-2

[3] Lillyman S , Bennett C . Providing a positive learning experience for international students studying at UK universities: a literature review. J Res Int Educ. 2014;13(1):63‐75.

[4] Luhrenberg P , Rahimi‐Nedjat RK , Sagheb K , Sagheb K , Al‐Nawas B . The efficiency of a learning software compared to e‐books in dental education. Eur J Dent. 2022 16(2):437‐442. 34905779 10.1055/s-0041-1735932 PMC9339934

[5] Kämmerer PW , Wolf JM , Buttchereit I , Frerich B , Ottl P . Prospective clinical implementation of optional implant treatment into pregraduate dental education‐mini implants for retention and support of mandibular overdentures. Int J Implant Dent. 2021;7(1):87. 34505196 10.1186/s40729-021-00371-6 PMC8429539

[6] Atay E , Hey J , Beuer F , Bose MWH , Schweyen R . Evaluation of the accuracy of fully guided implant placement by undergraduate students and postgraduate dentists: a comparative prospective clinical study. Int J Implant Dent. 2024;10(1):6. 38324168 10.1186/s40729-024-00526-1 PMC10850045

[7] Azarmehr I , Stokbro K , Bell RB , Thygesen T . Surgical navigation: a systematic review of indications, treatments, and outcomes in oral and maxillofacial surgery. J Oral Maxillofac Surg. 2017;75(9):1987‐2005. 28193444 10.1016/j.joms.2017.01.004

[8] Zhu WY , Choi WS , Wong MCM , Pu JJ , Yang WF , Su YX . The learning curve of computer‐assisted free flap jaw reconstruction surgery using 3D‐printed patient‐specific plates: a cumulative sum analysis. Front Oncol. 2021;11:737769. 34604076 10.3389/fonc.2021.737769 PMC8481918

[9] Beek DM , Baan F , Liebregts J , et al. A learning curve in 3D virtual surgical planned orthognathic surgery. Clin Oral Investig. 2023;27(7):3907‐3915. 10.1007/s00784-023-05013-2 PMC10329591 37083986

[10] Ackermann J , Baumann J , Pape J , et al. Factors influencing surgical performance and learning progress in minimally invasive surgery—results of an interdisciplinary multicenter study. Int J Surg. 2023;109(10):2975‐2986. 37462985 10.1097/JS9.0000000000000590 PMC10583955

[11] Bosse HM , Mohr J , Buss B , et al. The benefit of repetitive skills training and frequency of expert feedback in the early acquisition of procedural skills. BMC Med Educ. 2015;15:22. 25889459 10.1186/s12909-015-0286-5 PMC4339240

[12] Youssef BR , Söhnel A , Welk A , et al. RCT on the effectiveness of the intraligamentary anesthesia and inferior alveolar nerve block on pain during dental treatment. Clin Oral Investig. 2021;25(8):4825‐4832. 10.1007/s00784-021-03787-x PMC8342397 33527192

[13] Kämmerer PW , Adubae A , Buttchereit I , Thiem DGE , Daubländer M , Frerich B . Prospective clinical study comparing intraligamentary anesthesia and inferior alveolar nerve block for extraction of posterior mandibular teeth. Clin Oral Investig. 2018;22(3):1469‐1475. 10.1007/s00784-017-2248-2 29034443

[14] Kämmerer PW , Daubländer M . Methodical bias for comparison of periodontal ligament injection and local infiltration anesthesia for routine extractions in the maxilla. Ther Clin Risk Manag. 2018;14:591‐594. 29617011 10.2147/TCRM.S163732 PMC5870661

[15] Meechan JG . Supplementary routes to local anaesthesia. Int Endod J. 2002;35(11):885‐896. 12453016 10.1046/j.1365-2591.2002.00592.x

[16] Kämmerer P , Palarie V , Schiegnitz E , Ziebart T , Al‐Nawas B , Daubländer M . Clinical and histological comparison of pulp anesthesia and local diffusion after periodontal ligament injection and intrapapillary infiltration anaesthesia. J Pain Relief. 2012;1(10.4172):2167‐0846.1000108.

[17] Hochman MN , Friedman MJ , Williams W , Hochman CB . Interstitial tissue pressure associated with dental injections: a clinical study. Quintessence Int. 2006;37(6):469‐476. 16752703

[18] Girdler J . Dentapen versus traditional syringe infiltration—which LA technique is preferred by patients?. Evid Based Dent. 2022;23(3):100‐101. 36151280 10.1038/s41432-022-0811-4

[19] Kwak EJ , Pang NS , Cho JH , Jung BY , Kim KD , Park W . Computer‐controlled local anesthetic delivery for painless anesthesia: a literature review. J Dent Anesth Pain Med. 2016;16(2):81‐88. 28879299 10.17245/jdapm.2016.16.2.81 PMC5564086

[20] Aggarwal V , Singla M , Miglani S , Kohli S , Sharma V , Bhasin SS . Does the volume of supplemental intraligamentary injections affect the anaesthetic success rate after a failed primary inferior alveolar nerve block? A randomized‐double blind clinical trial. Int Endod J. 2018;51(1):5‐11. 28370327 10.1111/iej.12773

[21] Partido BB , Nusstein JM , Miller K , Lally M . Maxillary lateral incisor injection pain using the dentapen electronic syringe. J Endod. 2020;46(11):1592‐1596. 32763435 10.1016/j.joen.2020.07.029

[22] Brand HS , Tan LL , van der Spek SJ , Baart JA . European dental students' opinions on their local anaesthesia education. Eur J Dent Educ. 2011;15(1):47‐52. 21226806 10.1111/j.1600-0579.2010.00633.x

[23] Kanaa MD , Whitworth JM , Meechan JG . A prospective randomized trial of different supplementary local anesthetic techniques after failure of inferior alveolar nerve block in patients with irreversible pulpitis in mandibular teeth. J Endod. 2012;38(4):421‐425. 22414822 10.1016/j.joen.2011.12.006

[24] Shahi S , Rahimi S , Yavari HR , Ghasemi N , Ahmadi F . Success rate of 3 injection methods with articaine for mandibular first molars with symptomatic irreversible pulpitis: a CONSORT randomized double‐blind clinical trial. J Endod. 2018;44(10):1462‐1466. 30174101 10.1016/j.joen.2018.07.010

[25] Parirokh M , Sadr S , Nakhaee N , Abbott PV , Askarifard S . Efficacy of supplementary buccal infiltrations and intraligamentary injections to inferior alveolar nerve blocks in mandibular first molars with asymptomatic irreversible pulpitis: a randomized controlled trial. Int Endod J. 2014;47(10):926‐933. 24359138 10.1111/iej.12236

[26] Kämmerer PW , Schiegnitz E , von Haussen T , et al. Clinical efficacy of a computerised device (STA™) and a pressure syringe (VarioJect INTRA™) for intraligamentary anaesthesia. Eur J Dent Educ. 2015;19(1):16‐22. 24646115 10.1111/eje.12096

[27] Jing Q , Wan K , Wang XJ , Ma L . Effectiveness and safety of computer‐controlled periodontal ligament injection system in endodontic access to the mandibular posterior teeth. Chin Med Sci J. 2014;29(1):23‐27. 24698674 10.1016/s1001-9294(14)60019-5

[28] Kämmerer PW , Staedt H , Wesslau K , et al. The bevel effect: a prospective, randomized investigation into needle design in dental intraligamentary anesthesia. Clin Oral Investig. 2024;28(3):170. 10.1007/s00784-024-05546-0 38396049

[29] Chen LS , Nusstein J , Drum M , Fowler S , Reader A , Guo X . Effect of a combination of nitrous oxide and intraligamentary injection on the success of the inferior alveolar nerve block in patients with symptomatic irreversible pulpitis. J Endod. 2021;47(12):1890‐1895. 34492232 10.1016/j.joen.2021.08.013

[30] Zargar N , Shooshtari E , Pourmusavi L , Akbarzadeh Baghban A , Ashraf H , Parhizkar A . Anaesthetic efficacy of 4% articaine in comparison with 2% lidocaine as intraligamentary injections after an ineffective inferior alveolar nerve block in mandibular molars with irreversible pulpitis: a prospective randomised triple‐blind clinical trial. Pain Res Manag. 2021;2021:6668738. 34055121 10.1155/2021/6668738 PMC8131152

[31] Shabazfar N , Daubländer M , Al‐Nawas B , Kämmerer PW . Periodontal intraligament injection as alternative to inferior alveolar nerve block–meta‐analysis of the literature from 1979 to 2012. Clin Oral Investig. 2014;18(2):351‐358. 10.1007/s00784-013-1113-1 24077785

[32] Helmy RH , Zeitoun SI , El‐Habashy LM . Computer‐controlled intraligamentary local anaesthesia in extraction of mandibular primary molars: randomised controlled clinical trial. BMC Oral Health. 2022;22(1):194. 35596166 10.1186/s12903-022-02194-2 PMC9121608

[33] Anil O , Keskin G . Comparison of computer controlled local anesthetic delivery and traditional injection regarding disruptive behaviour, pain, anxiety and biochemical parameters: a randomized controlled trial. J Clin Pediatr Dent. 2024;48(1):120‐127. 10.22514/jocpd.2023.046 38239164

[34] Patil AN , Saurabh S , Pragya P , Aijazuddin A , Chandra S , Singh Chawla JP . Comparative assessment of perceived pain in children during palatal anesthesia using two injection techniques: an in vivo study. J Pharm Bioallied Sci. 2022;14(suppl 1):S503‐S506. 36110584 10.4103/jpbs.jpbs_71_22 PMC9469312

[35] Drum M , Reader A , Nusstein J , Fowler S . Successful pulpal anesthesia for symptomatic irreversible pulpitis. J Am Dent Assoc. 2017;148(4):267‐271. 28190451 10.1016/j.adaj.2017.01.002

[36] Pigg M , Nixdorf DR , Nguyen RH , Law AS . Validity of preoperative clinical findings to identify dental pulp status: a National Dental Practice‐Based Research Network study. J Endod. 2016;42(6):935‐942. 27118600 10.1016/j.joen.2016.03.016 PMC4884138

[37] Sánchez‐Garcés M , Arnabat‐Domínguez J , Camps‐Font O , Toledano‐Serrabona J , Guijarro‐Baude A , Gay‐Escoda C . Evaluation of student‐to‐student local anaesthesia administration at the University of Barcelona: a cross‐sectional study. Eur J Dent Educ. 2020;24(2):328‐334. 31981440 10.1111/eje.12503

[38] Wong G , Apthorpe HC , Ruiz K , Nanayakkara S . Student‐to‐student dental local anesthetic preclinical training: impact on students' confidence and anxiety in clinical practice. J Dent Educ. 2019;83(1):56‐63. 30600250 10.21815/JDE.019.007

[39] Al‐Shayyab MH . Periodontal ligament injection versus routine local infiltration for nonsurgical single posterior maxillary permanent tooth extraction: comparative double‐blinded randomized clinical study. Ther Clin Risk Manag. 2017;13:1323‐1331. 29070950 10.2147/TCRM.S143173 PMC5640402

[40] Ma L , Wan K , Jing Q , Kong LJ , Feng Z , Tian BB . Comparison of periodontal ligament anesthesia and submucosal infiltration anesthesia in healthy volunteers. Zhongguo Yi Xue Ke Xue Yuan Xue Bao. 2014;36(3):271‐276. 24997819 10.3881/j.issn.1000-503X.2014.03.008

[41] Nusstein J , Berlin J , Reader A , Beck M , Weaver JM . Comparison of injection pain, heart rate increase, and postinjection pain of articaine and lidocaine in a primary intraligamentary injection administered with a computer‐controlled local anesthetic delivery system. Anesth Prog. 2004;51(4):126‐133. 15675261 PMC2007494

[42] Pertot WJ , Déjou J . Bone and root resorption. Effects of the force developed during periodontal ligament injections in dogs. Oral Surg Oral Med Oral Pathol. 1992;74(3):357‐365. 1408000 10.1016/0030-4220(92)90076-3

[43] Iwanaga J , Choi PJ , Vetter M , et al. Anatomical study of the lingual nerve and inferior alveolar nerve in the pterygomandibular space: complications of the inferior alveolar nerve block. Cureus. 2018;10(8):e3109. 30338184 10.7759/cureus.3109 PMC6175254

[44] Schulz P , Sagheb K , Affeldt H , et al. Acceptance of e‐learning devices by dental students. Med 2 0. 2013;2(2):e6. 25075241 10.2196/med20.2767 PMC4084775

[45] Walton RE , Garnick JJ . The periodontal ligament injection: histologic effects on the periodontium in monkeys. J Endod. 1982;8(1):22‐26. 6948904 10.1016/S0099-2399(82)80312-9

[46] AlHindi M , Rashed B , AlOtaibi N . Failure rate of inferior alveolar nerve block among dental students and interns. Saudi Med J. 2016;37(1):84‐89. 26739980 10.15537/smj.2016.1.13278 PMC4724685

[47] Lee JS , Graham R , Bassiur JP , Lichtenthal RM . Evaluation of a local anesthesia simulation model with dental students as novice clinicians. J Dent Educ. 2015;79(12):1411‐1417. 26632295

[48] Corrêa CG , Machado M , Ranzini E , Tori R , Nunes FLS . Virtual reality simulator for dental anesthesia training in the inferior alveolar nerve block. J Appl Oral Sci. 2017;25(4):357‐366. 28877273 10.1590/1678-7757-2016-0386 PMC5595107

